# Draft Genome Sequence of a Multidrug-Resistant New Zealand Isolate of *Mycobacterium tuberculosis* Lineage 3

**DOI:** 10.1128/genomeA.01017-14

**Published:** 2014-10-16

**Authors:** Micheál Mac Aogáin, Bushra M. Johari, James E. Bower, Ronan F. O’Toole

**Affiliations:** a Department of Clinical Microbiology, School of Medicine, Trinity College Dublin, St. James’s Hospital, Dublin, Ireland; b LabPlus, Auckland City Hospital, Auckland, New Zealand; c Breathe Well Centre of Research Excellence, School of Medicine, University of Tasmania, Hobart, Tasmania, Australia

## Abstract

Multidrug resistance constitutes a threat worldwide to the management of tuberculosis (TB). We report the draft whole-genome sequence of a lineage 3 (East-African Indian) isolate of *Mycobacterium tuberculosis* which presented as multidrug resistant in New Zealand, and describe a number of single-nucleotide polymorphisms in genes relating to drug resistance.

## GENOME ANNOUNCEMENT

Tuberculosis (TB) is a leading cause of infectious mortality worldwide, killing in the region of 1.3 million people each year (1). There are an estimated 450,000 cases annually of multidrug-resistant (MDR)-TB, defined as resistant to rifampin and isoniazid (1). Phenotypic drug-susceptibility testing (DST) of MDR isolates can take several weeks to complete. Rapid determination of the drug-resistance profile of *Mycobacterium tuberculosis* complex (MTBC) isolates, through improvements in diagnostic technologies, would facilitate early optimal selection of anti-TB drugs in MDR cases.

Here, we applied next generation sequencing (NGS) for whole-genome analysis of drug-resistance mutations in a New Zealand clinical isolate of MDR-TB from 2011 which belongs to lineage 3 (East-African Indian) (2). Genomic DNA of the isolate (strain NZ3MDR1), was sequenced using an Illumina MiSeq instrument. A total of 2,642,010 paired-end reads were mapped to the *M. tuberculosis* strain H37Rv reference genome (accession no. AL123456.3) by Burrows-Wheeler alignment (3). This yielded an average read depth of 53-fold, covering 98.5% of the H37Rv genome. A consensus sequence was called using the SAMtools analysis suite (4), yielding a 4,329,366-bp draft assembly of 150 contigs. Single-nucleotide polymorphism (SNP) annotation was performed using snpEff (5) which identified a total of 1,320 SNPs in the assembled NZ3MDR1 genome with respect to H37Rv, of which 712 were non-synonymous.

A non-synonymous mutation was identified in the gene Rv0667 (*rpoB*) [tCg/tTg, S450L] which has previously been shown to have high specificity for rifampin phenotypic resistance (6). Two non-synonymous mutations were identified in the gene Rv1908c (*katG*), [aGc/aCc, S315T] and [cGg/cTg, R463L], the former having high specificity for isoniazid phenotypic resistance (6). These data confirm the genetic bases of the MDR phenotype exhibited by strain NZ3MDR1.

In terms of other first-line drugs, nonsynonymous mutations were detected in the genes Rv3793 (*embC*) [cGg/cAg, R738Q] and Rv3795 (*embB*) [gGc/gAc, G406D] although their specificities with respect to ethambutol resistance remain to be established. Nonsynonymous mutations were not detected in genes associated with pyrazinamide resistance, i.e., Rv2043c (*pncA*), Rv1630 (*rpsA*), and Rv3601c (*panD*) (7). This is in agreement with phenotypic DST results for this isolate which previously found susceptibility to ethambutol and pyrazinamide.

Regarding second-line drug resistance, SNPs were identified in Rv0006 (*gyrA*) [Gag/Cag, E21Q; aGc/aCc, S95T; gGc/gAc, G668D]. The S95T mutation has been demonstrated to have low specificity with respect to fluoroquinolone resistance (6) in agreement with the ofloxacin sensitivity of the NZ3MDR1 strain. In line with amikacin sensitivity, there were no SNPs identified in MTB000019 (*rrs*) of NZ3MDR1.

To our knowledge, this is the first published genome sequence of a lineage 3 MTBC isolate from New Zealand. The drug-resistance gene SNP data generated correlate with earlier phenotypic DST data but were obtained within a considerably shorter turn-around-time. In addition, deletions were detected in known MTBC virulence and immune modulatory genes in NZ3MDR1, the significance of which will be investigated further. This underlines the value that NGS could offer clinical diagnostics in the routine characterization of TB isolates.

### Nucleotide sequence accession number.

This whole-genome shotgun project has been deposited at DDBJ/EMBL/GenBank under the accession no. CCSJ00000000.
